# Unraveling the Complexities of Small Intestinal Bacterial Overgrowth

**DOI:** 10.3390/medicina62030485

**Published:** 2026-03-04

**Authors:** Enrico Capuano, Angelo Del Gaudio, Enrico Celestino Nista, Sara Sofia De Lucia, Antonio Pellegrino, Giuseppe Cuccia, Angela Saviano, Carmine Petruzziello, Veronica Ojetti

**Affiliations:** 1 Department of Medical and Surgical Sciences, Fondazione Policlinico Universitario A. Gemelli IRCCS, 00168 Rome, Italy; enrico.capuano01@icatt.it (E.C.); angelo.delgaudio@guest.policlinicogemelli.it (A.D.G.); sarasofia.delucia@guest.policlinicogemelli.it (S.S.D.L.);; 2 Ospedale Verris delli Ponti Scorrano ASL Lecce, 73020 Scorrano, Italy; 3 Department of Emergency, Anesthesiological, and Reanimation Sciences, Università Cattolica Sacro Cuore, Fondazione Policlinico Universitario A. Gemelli IRCCS, 00168 Rome, Italy; angela.saviano@policlinicogemelli.it; 4 Ospedale San Carlo di Nancy, GVM Research, 00165 Rome, Italy; 5 Department of Internal Medicine, UniCamillus International Medical University of Rome, 00131 Rome, Italy

**Keywords:** SIBO, gut microbiota, small intestine, probiotics

## Abstract

This narrative review explores the intricate dynamics of Small Intestinal Bacterial Overgrowth (SIBO), a condition marked by an excessive growth of bacteria in the small intestine. We delve into the multifaceted etiology of SIBO, including its associations with gastrointestinal disorders, motility issues, and alterations in gut architecture. The review examines current diagnostic methodologies, emphasizing the distinctions between breath testing and direct aspiration techniques. Furthermore, we assess the various treatment approaches, ranging from antibiotics and dietary modifications to emerging therapeutic options. By synthesizing the latest research findings, we aim to enhance understanding of SIBO’s implications for gut health and provide insights into effective management strategies.

## 1. Introduction

The historical trajectory of Small Intestinal Bacterial Overgrowth (SIBO) provides crucial insights into its persistent diagnostic challenges. The condition was first systematically characterized by Barker and Hummel in 1939 as “blind loop syndrome”—a malabsorption disorder resulting from bacterial colonization in anatomically altered small intestines [1]. Subsequent work by Card in 1959 established bacterial overgrowth as the common pathological mechanism underlying diverse clinical scenarios, including intestinal strictures and diverticulosis [2]. Badenoch’s comprehensive 1960 classification further refined the clinical spectrum, distinguishing between postoperative “surgical” cases and “medical” presentations featuring megaloblastic anemia and steatorrhea. His detailed documentation of metabolic consequences, including coagulopathy from vitamin K deficiency, bone disease from vitamin D malabsorption, and complex anemia patterns from combined B12 and iron deficiency—established the classic malabsorptive phenotype that defined early understanding of the disorder [3].

The contemporary definition recognizes SIBO as a disruption of the physiological microbial gradient, where bacterial density exceeding 10^3^ CFU/mL in the small intestine produces gastrointestinal symptomatology. This represents a fundamental disturbance of the normal ecosystem, where proximal small intestinal bacterial counts are typically maintained below 10^3^ CFU/mL, standing in stark contrast to colonic concentrations that may reach 10^11^ CFU/mL [4,5,6]. The failure of host defense mechanisms permits uncontrolled proliferation of predominantly colonic bacteria, which ferment dietary carbohydrates to produce gas. This pathological fermentation process directly underlies the characteristic clinical manifestations of bloating, abdominal pain, and osmotic diarrhea through malabsorptive mechanisms [7].

The evolution of SIBO conceptualization has paralleled diagnostic advancements. While the historical gold standard of duodenal aspirate culture remains specific, its clinical utility is limited by invasiveness, cost, and contamination risk [8], establishing breath testing as the predominant contemporary modality [9]. The diagnostic principle relies on detecting gases produced by bacterial fermentation of substrates—typically glucose or lactulose. However, substrate selection introduces significant diagnostic variability: glucose’s proximal absorption may yield false negatives for distal overgrowth, while lactulose’s passage to the colon can produce confounding results [10]. Particularly important is the recognition that these gases correlate with specific clinical phenotypes—methane with constipation-predominant symptoms [11] and hydrogen sulfide with diarrhea-predominant manifestations [11]—representing [12].

Therapeutic management remains challenging due to SIBO’s multifactorial etiology and recurrence rates. The cornerstone triple strategy encompasses addressing underlying causes, providing nutritional support, and reducing bacterial burden primarily with antibiotics like rifaximin. However, antibiotic therapy is frequently complicated by microbial resistance, adverse effects, and dysbiosis, often necessitating repeated courses and prompting investigation of alternative approaches [13]. Among emerging strategies, innovative use of probiotics, prebiotics, and postbiotics show considerable promise for their ability to beneficially modulate the gut microenvironment [14,15,16]. Dietary intervention, particularly the Low FODMAP Diet (LFD), represents another key management strategy. By restricting fermentable carbohydrates, the LFD effectively reduces gas production, osmotic diarrhea, and bloating, thereby alleviating symptoms and improving quality of life [17].

The current understanding of SIBO continues to evolve with advancements in microbiome science, moving beyond the oversimplified concept of colonic flora migration toward recognizing specific opportunistic organisms. The integration of next-generation sequencing with refined breath testing capabilities represents a paradigm shift from the historical anatomical model to a sophisticated, microbiome-based understanding of this complex and challenging disorder [18]. This narrative review, drawing upon the most recent scientific literature on the topic, aims to synthesize this evolving landscape. It will critically examine the contemporary pathophysiology, diagnostic complexities, and therapeutic paradigms of SIBO to inform clinical practice and highlight future research directions.

## 2. Pathogenesis of SIBO

The pathogenesis of SIBO is multifactorial, first of all, the protective mechanisms—including intestinal peristalsis, the antimicrobial activity of gastric acid and pancreaticobiliary secretions, and local immune surveillance—work in concert to preserve a healthy proximal-to-distal microbial gradient. The compromise of any component, whether through dysmotility, hypochlorhydria, anatomical alterations, or immune deficiency, disrupts this delicate balance and permits pathological bacterial colonization [19]. This underlying pathophysiology accounts for SIBO’s well-documented associations with a spectrum of disorders, such as irritable bowel syndrome, inflammatory bowel disease, chronic pancreatitis, and connective tissue diseases. However, the considerable epidemiological overlap with these conditions, combined with persistent diagnostic challenges, continues to obscure a precise determination of its true prevalence and overall clinical impact.

### 2.1. Altered Motility

Impaired intestinal motility is a primary mechanism in the development of SIBO, largely due to the failure of the migrating motor complex (MMC). This cyclical inter-digestive mechanism is essential for cleaning the bowel and preventing bacterial stasis [20]. The resulting dysmotility creates an environment that favors colonization by colonic flora [21,22], with recent wireless motility capsule studies directly linking SIBO development to slowed small intestinal transit specifically, rather than delayed gastric emptying [23]. The clinical relevance of this mechanism is well-established in conditions like diabetic autonomic neuropathy, where patients face a 2.91-fold increased SIBO risk attributed to hyperglycemia and inflammation-induced enteric neural damage [24,25]. SIBO prevalence rises to 40% in diabetic patients with diarrhea [26], a significant finding given that gastrointestinal symptoms affect 50–70% of individuals with diabetes. The documented delays in gastric emptying and intestinal dysmotility in this population not only drive SIBO development but also adversely impact glycemic control [27,28]. Breath testing studies have confirmed SIBO rates of 28–60%, which correlate with prolonged gastroparetic symptoms and cardiovascular autonomic neuropathy [29,30]. A parallel pathophysiological pathway is observed in systemic sclerosis were gastrointestinal involvement affects over 80% of patients [31]. Here, dysmotility involves gastrointestinal fibrosis and autoantibodies against muscarinic receptors, leading to a SIBO prevalence of 43–56% [32,33,34]. The clinical significance of this association is underscored by the significant symptomatic relief achieved after successful SIBO eradication with antibiotic therapy, particularly rifaximin [32], while evidence supporting prokinetics or probiotics remains limited. Moreover, recent pilot data suggest potential benefit from *Saccharomyces boulardii* for managing gastrointestinal symptoms and SIBO in systemic sclerosis, either alone or in combination with metronidazole [35]. The spectrum of motility-related disorders predisposing to SIBO further extends to amyloidosis [36], myotonic dystrophy [37], multiple sclerosis [38], and chronic intestinal pseudo-obstruction [39]. A notable structural manifestation is jejunal diverticulosis, where underlying neurogenic or myogenic motility disorders drive the formation of large, multiple diverticula. These structural anomalies, are present in 0.07–2% of the population, more common in men over 60, and significantly predispose to SIBO, as confirmed by several case reports [40]. Consequently, SIBO should be a key diagnostic consideration in patients with conditions that affect motility and who present with unexplained gastrointestinal symptoms.

### 2.2. Immune System Dysregulation

Compromised immunity, whether hereditary, acquired, or related to dysregulation, constitutes a significant risk factor for SIBO. The Gut-Associated Lymphoid Tissue (GALT), including Peyer’s patches and secretory IgA, is crucial for maintaining intestinal microbial homeostasis by regulating bacterial load and diversity [41]. A breach in these defenses predisposes individuals to bacterial overgrowth [42,43], a connection evident in conditions ranging from hypogammaglobulinemia and HIV infection [44] to immune-mediated disorders like celiac disease and inflammatory bowel disease. Within celiac disease, SIBO is a frequent complication, with prevalence rates of 9–55%. It is particularly common in patients with persistent symptoms despite a gluten-free diet or those with concomitant lactose intolerance [45,46,47]. A systematic review and meta-analysis concluded that while SIBO prevalence was not significantly higher in non-responsive celiac disease, antibiotic therapy was highly effective in resolving symptoms and normalizing breath tests in confirmed cases [48]. Similarly, a substantial SIBO overlap exists in Crohn’s disease, affecting 16.8–25% of patients, including those in endoscopic remission [49]. This risk escalates significantly following surgical resection—particularly of the ileo-cecal valve—or in the presence of enteric fistulae, which disrupt normal intestinal anatomy and bacterial containment [50,51]. Clinically, SIBO can mimic an acute Crohn’s flare with symptoms like increased bowel movements and weight loss, necessitating careful differential diagnosis [52]. The incidence is notably higher in Crohn’s disease than in ulcerative colitis, especially among older patients and during active disease phases [53]. Systemic immune compromise further exacerbates SIBO risk, as demonstrated in AIDS, where vagal neuropathy delays gastric emptying and intestinal transit, creating a favorable environment for bacterial proliferation [54]. Studies combining intestinal transit analysis and breath testing in HIV patients have identified this vagal dysfunction as a significant risk factor, with SIBO present in over half of the participants studied [54].

### 2.3. Anatomical Alterations

Surgically altered gastrointestinal anatomy represents a major risk factor for SIBO, primarily through mechanisms that create blind loops, promote intestinal stasis, and compromise natural defense barriers. The foundational understanding of this link originated from observations in post-gastrectomy patients, where the loss of gastric acid—a potent bacteriostatic agent—plays a central role [55]. This principle is starkly illustrated by Roux-en-Y gastric bypass surgery, a procedure that creates a functional blind loop and bypasses the acidic gastric environment, leading to notably high SIBO incidences of 43–73.4% [56]. This association extends to other common procedures, including cholecystectomy, which carries a SIBO prevalence of approximately 45%, potentially mediated by altered bile acid homeostasis [57]. Even non-GI surgeries like hysterectomy demonstrate a significant link, with a 41.1% prevalence likely resulting from adhesion-related disruption of intestinal motility [58]. This surgical predisposition is especially relevant in inflammatory bowel disease (IBD), where meta-analyses confirm significantly higher SIBO rates in IBD patients compared to controls, with risk being most markedly elevated in those with surgically managed Crohn’s disease [59]. Following surgical intervention, prevalence reaches 31.8% compared to 19.2% in non-operated counterparts, a difference strongly associated with resection of the ileocecal valve [60]. The ileocecal valve’s critical function as a barrier is further highlighted by studies demonstrating that structural incompetence, characterized by low resting pressures, facilitates colonic bacterial reflux into the small intestine and is a well-documented independent risk factor for SIBO [61]. Collectively, these findings underscore how surgical interventions, while addressing specific disease processes can inadvertently create milieus of stasis and dysbiosis that foster bacterial overgrowth, thereby complicating long-term patient management.

### 2.4. Altered Gastrointestinal Secretion

The integrity of gastrointestinal secretory functions constitutes a fundamental defense mechanism against bacterial overgrowth. When these protective secretions are diminished, the loss of inherent antibacterial activity fosters a permissive environment for microbial proliferation in the small intestine. A clinically significant instance of this disruption is medication-induced hypochlorhydria resulting from prolonged proton pump inhibitor (PPI) therapy, which effectively neutralizes the gastric acid barrier. This pathophysiological alteration is reflected in meta-analytic data, demonstrating a nearly two-fold increase in SIBO risk (OR 1.7–1.71), a correlation further supported by the higher prevalence of PPI use among patients with culture-positive duodenal aspirates [62,63].

Beyond gastric acid, pancreatic exocrine secretion plays a dual protective role. In chronic pancreatitis, exocrine pancreatic insufficiency (EPI) predisposes to SIBO not only through the loss of antimicrobial peptides but also via impaired stimulation of intestinal motility [64]. This core defect is frequently compounded by associated factors such as motility disorders and medication effects. Consequently, SIBO is markedly prevalent in this population, with meta-analyses indicating a pooled rate of 36–38%, representing a four-fold elevated risk [65,66]. Clinicians should maintain a high index of suspicion for SIBO in patients with chronic pancreatitis who exhibit severe markers like diabetes, chronic opiate use, or specific nutritional deficiencies, particularly when symptoms persist despite adequate enzyme replacement [67,68].

Transitioning to the hepatobiliary system, bile acids emerge as critical regulators of the intestinal microbial landscape. Deficiency in bile acid secretion or function, common in chronic liver disease, removes a key innate antimicrobial defense. The prevalence of SIBO escalates in parallel with liver disease severity, reaching 41.2% in cirrhosis and correlating with Child-Pugh classification [69]. Systematic reviews substantiate a substantially elevated risk (OR 6.7), with prevalence peaking at 57.7% in decompensated cirrhosis accompanied by complications like portal hypertension [70]. The pathophysiological interplay in cirrhosis involves a triad of portal hypertension, an altered luminal environment, and significant dysmotility, leading to SIBO in 50–60% of patients. This association is clinically paramount, as SIBO is a well-documented risk factor for spontaneous bacterial peritonitis (SBP), evidenced by a 70% prevalence in cirrhotics with SBP compared to 20% without [71]. Even in the absence of advanced liver disease, gallbladder dysfunction—such as in gallstone pathology—can impair the postprandial release of bile, creating a local deficiency that facilitates bacterial overgrowth [72].

This connection between SIBO and hepatic health extends prominently to non-alcoholic fatty liver disease (NAFLD), where SIBO prevalence is approximately 50%, significantly exceeding that of healthy controls [73,74]. Here, intestinal dysbiosis is implicated in NAFLD pathogenesis via a self-perpetuating cycle. SIBO-related dysbiosis compromises intestinal barrier integrity, leading to increased permeability and systemic endotoxemia, notably from lipopolysaccharide (LPS) [74]. The altered microbiota also produces hepatotoxic metabolites, including endogenous ethanol and disturbed bile acid species, which translocate to the liver to promote inflammation and steatosis [75,76]. A hallmark of this dysbiosis in NAFLD is a bloom of Gram-negative bacteria, reduced microbial diversity, and elevated synthesis of pro-inflammatory molecules like LPS and trimethylamine. Concurrently, metabolic disturbances such as impaired insulin/IGF1 signaling and hyperglycemia further disrupt epithelial function, creating parallel pathways that synergistically drive the intestinal hyperpermeability central to disease progression [77]. Thus, disrupted secretion—from the stomach to the biliary tree—creates a permissive milieu that not only predisposes to SIBO but also actively contributes to systemic and hepatic metabolic dysfunction.

## 3. The Association Between Irritable Bowel Syndrome (IBS) and SIBO

The clinical and pathophysiological relationship between irritable bowel syndrome (IBS) and Small Intestinal Bacterial Overgrowth (SIBO) remains a subject of considerable scientific debate. While substantial symptomatic overlap exists between these conditions, fundamental questions regarding causality and optimal diagnostic approaches persist despite extensive investigation. Epidemiological studies consistently demonstrate remarkable co-prevalence, with research indicating that 30–85% of patients meeting IBS diagnostic criteria simultaneously present with SIBO [78,79,80]. A comprehensive meta-analysis by Ghoshal et al. (2022) consolidates this broad range, reporting a pooled prevalence of SIBO in IBS patients of 49% based on breath testing, with significant geographical variation observed, being higher in developing (57%) compared to developed (41%) nations [81]. From the opposite perspective, studies examining SIBO cohorts find that a majority fulfill the Rome IV criteria for IBS, underscoring the bidirectional nature of this clinical overlap [82]. This substantial overlap has stimulated several competing theoretical frameworks.

One perspective posits SIBO as the initial pathological event, with subsequent IBS symptoms emerging as a direct consequence of bacterial overgrowth. Mechanistically, this is proposed to occur via several pathways: bacterial fermentation of carbohydrates leading to gas production (hydrogen, methane, hydrogen sulfide) and osmotic diarrhea; deconjugation of bile acids resulting in fat maldigestion and secretory diarrhea; and direct mucosal injury triggering low-grade inflammation and visceral hypersensitivity [83,84,85,86]. Conversely, alternative hypotheses suggest that pre-existing intestinal motility abnormalities—a recognized feature of IBS, particularly the phase III of the migrating motor complex (MMC)—create environmental conditions favorable for subsequent bacterial overgrowth development [83]. This impaired “housekeeper” function allows for bacterial stasis and proliferation. Furthermore, underlying immune dysfunction, including reduced IgA secretion or subtle alterations in innate immunity, may compromise mucosal defense, facilitating bacterial colonization [84,85,86]. A third, more evident perspective advocates for maintaining clear diagnostic separation between these entities as distinct clinical conditions despite symptomatic similarities, with some researchers questioning whether SIBO plays any meaningful role in IBS pathogenesis [85].

The diagnostic landscape is complicated by significant methodological concerns, leading to a high risk of diagnostic overlap and misclassification. Breath testing, despite widespread clinical adoption, demonstrates considerable accuracy variability. Initial investigations reported strikingly high rates of abnormal lactulose breath test results (84%) among IBS patients [87], while more recent studies employing duodenal aspirate cultures with modified diagnostic thresholds (≥10^3^ CFU/mL) have yielded a more nuanced understanding [88]. The lack of a standardized diagnostic gold standard is a central issue; culture-based methods are invasive and may miss fastidious or mucosal-adherent bacteria, while breath tests are confounded by rapid oro-cecal transit, colonic fermentation, and arbitrary cutoff values [89]. This diagnostic uncertainty directly inflates prevalence estimates and complicates the interpretation of therapeutic trials. The recognition of distinct intestinal gas production profiles has substantially advanced pathophysiological comprehension, with methane-predominant patterns showing robust association with constipation-predominant IBS (IBS-C), while hydrogen and hydrogen sulfide profiles demonstrate stronger connections to diarrheal-predominant IBS (IBS-D) [89]. These differential gas production patterns reflect fundamental variations in microbial communities, with methanogenic archaea, including *Methanobrevibacter smithii,* predominating in IBS-C, while hydrogen sulfide-producing species such as *Fusobacterium* and *Desulfovibrio* appear enriched in IBS-D patients [90].

Contemporary molecular methodologies enable increasingly detailed characterization of small intestinal microbial composition. Investigations utilizing 16S ribosomal RNA gene sequencing reveal that SIBO patients exhibit distinct microbial patterns characterized by reduced overall diversity (α-diversity), increased Proteobacteria abundance, and decreased Firmicutes representation. This dysbiotic signature often features an expansion of facultative anaerobes at the expense of obligate anaerobes, indicating a fundamental shift in the small intestinal ecological niche [87,88,89,90,91]. Importantly, the concept of microbial “dysbiosis”—defined as compositional deviation from healthy control patterns—may provide better differentiation between symptomatic and asymptomatic individuals than conventional SIBO diagnostic criteria based solely on bacterial density [89].

Species including *Escherichia coli* and various *Klebsiella* species dominate the duodenal microbiome in symptomatic SIBO cases [18], remarkably consistent with historical observations regarding their role in malabsorption syndromes [92].

The symptomatic improvement observed following antibiotic treatment offers compelling, albeit indirect, evidence for a bacterial component in the pathophysiology of IBS. Rifaximin, in particular, demonstrates statistically significant—though clinically modest—efficacy in non-constipated IBS. It is noteworthy that its therapeutic action may be mediated through the modulation of fermentation processes within the colon, rather than a direct effect on the small intestine [93,94,95,96]. Other antibiotic regimens, such as norfloxacin and metronidazole, also show efficacy, supporting the bacterial hypothesis. However, high relapse rates (up to 44% within 9 months post-rifaximin) suggest that antibiotic therapy addresses a consequence rather than the root cause of the dysbiosis or motility disorder. Calculated numbers needed to treat range between 10.1 for bloating-specific improvement and 11.1 for global symptom response [97], indicating that only a specific patient subset derives meaningful benefit. Interestingly, while pre-treatment breath test positivity appears to predict therapeutic response to rifaximin, post-treatment test normalization does not reliably correlate with symptomatic improvement [97,98]. This dissociation suggests that symptoms may be driven by functional metabolic changes or by bacteria not fully eradicated, rather than by the quantitative bacterial load alone [8].

## 4. Clinical Phenotypes and Systemic Sequelae of SIBO

SIBO is characterized by two primary clinical presentations. The first, “classic” SIBO, results directly from bacterial overgrowth causing overt malabsorption, evidenced by steatorrhea, nutrient deficiencies, and protein-losing enteropathy; its diagnosis is often confirmed by a positive response to antibiotic therapy [99]. In contrast, the second, “expanded” phenotype features functional symptoms that mirror those of IBS, such as bloating and abdominal discomfort, but without significant malabsorption. The causal role of SIBO in this context remains debated, as diagnostic ambiguities and variable treatment responses make it challenging to determine if it is a primary etiology, a secondary consequence, or simply an epiphenomenon of the underlying disorder [100]. The pathophysiological cascade begins with mucosal damage and a reduction in brush border enzymes, which impairs carbohydrate digestion [101]. The subsequent fermentation of these undigested substrates by bacteria generates gases, including hydrogen and methane. These act as direct luminal irritants, provoking bloating, flatulence, and distension, with methane being specifically linked to constipation-predominant symptoms [102]. Parallel bacterial deconjugation of bile acids leads to fat malabsorption, causing steatorrhea and deficiencies in fat-soluble vitamins. The resulting free bile salts also have a pro-secretory effect on the colon, contributing to diarrhea [103]. Chronic SIBO can lead to systemic complications. Nutrient competition, particularly for vitamin B12, may result in megaloblastic anemia and polyneuropathy. Furthermore, persistent inflammation and mucosal injury increase intestinal permeability, facilitating the translocation of bacterial endotoxins into the circulation. This process can stimulate the production of neuroactive compounds like D-lactic acid, which can also precipitate neurological symptoms such as confusion [104]. D-lactic acidosis, a severe neurologic complication, occurs predominantly in patients with short bowel syndrome and an intact colon. Here, lactobacilli overgrowth ferments malabsorbed carbohydrates, producing D-lactic acid, a metabolite humans cannot efficiently clear. Its absorption can lead to characteristic intoxication-like encephalopathy, managed by correcting the acidosis, administering antibiotics, and implementing a dietary strategy that reduces simple carbohydrate intake [105]. Finally, the state of sustained immune activation is hypothesized to connect SIBO to various extra-intestinal disorders, including fibromyalgia and spondyloarthropathy, illustrating a potential gut-systemic axis of disease [106,107]. Other clinical manifestations may include skin lesions (e.g., rosacea), arthralgias, and lower extremity edema, the latter often having a multifactorial etiology involving anemia, hypoproteinemia, and specific nutrient deficiencies (see Table 1).

## 5. Diagnosis of Small Intestinal Bacterial Overgrowth

The diagnosis of SIBO presents a persistent clinical challenge due to the absence of a single, universally standardized test. Clinical suspicion should arise in patients presenting with non-specific gastrointestinal symptoms—particularly bloating, abdominal discomfort, and altered bowel habits—especially when predisposing conditions such as motility disorders, anatomical abnormalities, or malabsorption syndromes are present [6]. While physical examination and routine laboratory tests often yield non-specific findings, they may reveal abdominal distension, signs of nutrient deficiencies (including latent tetany, polyneuropathy, or rosacea), anemia, decreased vitamin B12 levels, and other indicators of malnutrition. Culture of duodenal or jejunal aspirate remains the acknowledged diagnostic gold standard. This invasive procedure involves obtaining intestinal fluid during endoscopic examination, followed by quantitative aerobic and anaerobic culturing using diagnostic thresholds of either >10^3^ or ≥10 r CFU/mL [7]. The method provides direct evidence of bacterial overgrowth and enables specific pathogen identification, offering considerable diagnostic accuracy [108]. However, it faces substantial limitations, including invasiveness, high cost, prolonged processing time, and patient discomfort. Major drawbacks encompass poor reproducibility, lack of standardization, and high technical demands [109]. Crucially, its reliability is compromised by the substantial proportion of unculturable gut bacteria and susceptibility to oropharyngeal contamination. Furthermore, since aspiration is typically limited to the proximal small intestine, it may miss distal ileal overgrowth [108]. These constraints restrict routine clinical application, establishing non-invasive breath testing as the preferred first-line screening modality despite its lower specificity. Hydrogen and methane breath testing serves as the primary non-invasive method for diagnosing both SIBO and intestinal methanogen overgrowth (IMO). The diagnostic principle relies on the exclusive bacterial and archaeal production of these gases through carbohydrate fermentation. While this process normally occurs predominantly in the colon, it pathologically extends to the small intestine in SIBO and IMO. A portion of the produced gases diffuses into the bloodstream and is excreted via pulmonary ventilation, enabling quantitative measurement in exhaled breath. Test accuracy demands rigorous patient preparation to minimize external confounding factors. Standardized protocols, such as those established by the North American Consensus, are essential. These mandate an 8–12 h fasting period to establish a reliable baseline, with a 12 h fast considered optimal for minimizing residual food fermentation [10,110]. The preceding day’s diet requires strict control, excluding complex carbohydrates, dairy, fermentable substrates (FODMAPs), and dietary fiber, all known to elevate baseline hydrogen and methane levels [111]. Medication management is equally critical, necessitating a four-week washout for antibiotics and one week for probiotics, laxatives, proton pump inhibitors, NSAIDs, and prokinetic agents [64,110,111]. On test day, patients must avoid smoking (which can cause transient hydrogen spikes) and strenuous activity (which alters gut motility and ventilation), maintaining sedentary conditions throughout the procedure [111,112,113]. Importantly, baseline hydrogen levels exceeding 16–20 ppm typically invalidate the test, necessitating retesting after stricter preparation [10,114]. For test administration, standard substrate dosages are 10 g of lactulose for the lactulose breath test (LBT) and 75 g of glucose [114]. Interpretation varies by substrate: a glucose test is considered positive with a hydrogen increase ≥12 ppm within 120 min, while a lactulose test requires either a biphasic pattern or an early hydrogen rise ≥12 ppm. A key diagnostic concept establishes that a methane increase of 5 ppm within 90 min is biologically equivalent to a 20 ppm hydrogen increase [10]. Simultaneous measurement of both gases is strongly recommended for comprehensive diagnostic accuracy. The distinct condition of IMO, characterized by methane-producing archaea, can be efficiently diagnosed using a single fasting methane measurement (SMM) of ≥10 ppm, demonstrating high specificity according to the North American Consensus [10]. This aligns with 2020 ACG guidelines defining IMO by a hydrogen rise ≥20 ppm coupled with methane ≥10 ppm within 90 min [7]. Clinically, IMO is associated with slowed intestinal transit and constipation, distinguishing it from hydrogen-dominant SIBO. Beyond conventional breath testing, innovative diagnostic modalities continue to emerge. Telemetric gas-sensing capsules measure intraluminal hydrogen, oxygen, and carbon dioxide concentrations, providing location-specific data that can pinpoint small intestinal fermentation sites, unlike breath tests that reflect whole-gut activity [115]. The Smart Capsule Bacteria Detection System (SCBDS) enables targeted duodenal fluid sampling with on-board bacterial analysis, demonstrating strong agreement (82–92%) with traditional culture methods [116]. Molecular techniques like 16S rRNA gene sequencing of duodenal aspirates offer detailed microbial profiling, revealing that SIBO associates with reduced α-diversity and characteristic taxonomic shifts, including increased Streptococcus and decreased Bacteroides abundance [117,118]. The scintigraphy-lactulose hydrogen breath test (ScLBT) represents another accurate diagnostic tool, though its equivalence to simpler tests requires further validation [119]. Historically, alternative methods analyzing jejunal short-chain fatty acids, serum bile acids, or urinary markers have been explored but lack sufficient reliability for routine clinical applications [5,120]. In settings where diagnostic testing is unavailable, an empiric trial with rifaximin for 7–10 days may be considered. While symptom resolution may support a SIBO diagnosis, it does not provide definitive proof, just as a positive test alone cannot conclusively establish causality between bacterial overgrowth and patient symptoms [5]. In conclusion, despite their established clinical utility, breath tests and emerging methodologies face significant challenges due to global standardization deficiencies, resulting in variable protocols and diagnostic criteria. The development of unified methodological standards remains an urgent priority to enhance diagnostic consistency and reliability in clinical practice.

## 6. Treatment of Small Intestinal Bacterial Overgrowth

The management of SIBO requires a structured, multi-component strategy centered on three fundamental pillars: the eradication of bacterial overgrowth, the identification and correction of nutritional deficiencies, and the treatment of underlying predisposing conditions where possible. Given that many underlying etiologies—such as visceral myopathies or multiple jejunal diverticula—are not readily reversible, pharmacological intervention with antibiotics remains the cornerstone of clinical management.

### 6.1. Antibiotic Therapy

The primary goal of antibiotic therapy is not the complete sterilization of the gut, which is neither feasible nor desirable, but rather a qualitative modification of the microbiota to achieve a composition that resolves clinical symptoms. While susceptibility testing of duodenal aspirates represents an ideal, it is often impractical due to the polymicrobial nature of overgrowth, necessitating empirical treatment. The non-absorbable rifamycin derivative rifaximin is the most extensively studied first-line agent, favored for its broad-spectrum activity and excellent safety profile, with minimal systemic absorption and negligible impact on colonic microbiota [121]. Its efficacy is established across doses ranging from 800 mg/day for four weeks to 1200–1650 mg/day for 7–14 days, showing superiority over metronidazole in some trials [122,123]. Higher doses correlate with improved breath test normalization rates [123,124], and meta-analyses confirm an approximate 70% eradication rate linked to symptomatic improvement [125].

For methane-predominant SIBO, now classified as Intestinal Methanogen Overgrowth (IMO), alternative strategies are required due to archaeal resistance. Combination therapy with rifaximin and neomycin yields the most significant symptomatic improvement, outperforming either agent alone [126]. Other antibiotics with demonstrated utility include quinolones (e.g., norfloxacin, ciprofloxacin), nitroimidazoles (metronidazole), and amoxicillin-clavulanic acid. In Crohn’s disease patients with SIBO, both metronidazole and ciprofloxacin achieve high breath test normalization rates [60]. Emerging evidence suggests that rotating between different antibiotic classes may yield higher remission rates than single-agent therapy and improve quality of life [127]. However, recurrence remains a major challenge, with rates reaching 44% within nine months of a single course [128]. For recurrent cases, long-term strategies such as cyclic or continuous rotating antibiotic regimens may be considered, albeit with careful risk-benefit assessment.

### 6.2. Adjunctive and Non-Antibiotic Therapies

Probiotics: The role of probiotics is complex and context-dependent. *Saccharomyces boulardii*, resistant to concurrent antibiotics, is the most studied. It enhances eradication rates and alleviates symptoms like bloating and diarrhea when combined with metronidazole or rifaximin/neomycin [35,129]. In cirrhosis, *S. boulardii* or specific bacterial blends reduce SIBO prevalence and complications like hepatic encephalopathy [130,131].

Meta-analyses support specific strains (e.g., *Lactobacillus rhamnosus*, *Bifidobacterium longum*) for improving decontamination rates and reducing abdominal pain, though effects on bowel frequency are limited [132,133,134]. Benefits are not universal, however, as probiotics show limited efficacy post-Roux-en-Y gastric bypass and carry risks of exacerbating symptoms or, rarely, causing D-lactic acidosis [135,136]. This underscores the need for cautious, strain-specific application [13,136,137].

Dietary Modifications: Dietary intervention is a key adjunctive strategy, primarily aimed at reducing fermentable substrates. The most evidence-supported approach is the Low FODMAP diet, which reduces breath hydrogen concentrations and symptom burden [138]. Professional guidance is crucial for its implementation to avoid long-term reduction in beneficial microbes like *Bifidobacterium* and *Akkermansia muciniphila* [139,140,141,142,143,144]. Alternative diets rich in complex carbohydrates and fiber may promote a beneficial microbiota [140]. Distinct dietary patterns, such as higher buckwheat and poultry intake, characterize treatment-resistant cases [141]. For severe, refractory SIBO, a 14-day elemental diet can be highly effective (85% response) by depriving bacteria of nutrients, though its use is limited by cost and palatability [142]. It is critical to recognize that dietary changes primarily manage symptoms without correcting the underlying pathophysiology [143]. Nutritional Support and Systemic Considerations: Nutritional support is indispensable, often involving lactose restriction and correction of deficiencies in fat-soluble vitamins (A, D, E, K), vitamin B12, calcium, and magnesium. In refractory cases, a comprehensive re-evaluation is essential to identify overlapping conditions like lactase deficiency or other food intolerances, which may necessitate concurrent long-term dietary management [145]. Emerging research highlights systemic dimensions of SIBO, including altered tryptophan metabolism via the kynurenine pathway, linked to inflammation and mood disorders. Rifaximin treatment can normalize these pathways while improving both gastrointestinal and psychiatric symptoms, underscoring the gut–brain axis involvement in SIBO [137].

## 7. Conclusions

SIBO endures as a sophisticated clinical entity, pathologically defined by a loss of the physiological microbial gradient, where bacterial density in the small intestine exceeds 10^3^ CFU/mL, thereby inciting gastrointestinal symptomatology. Its inherently multifactorial pathogenesis arises from a confluence of compromised host defenses, including dysmotility (e.g., MMC failure), anatomical alterations (e.g., blind loops, ileocecal valve dysfunction), deficient secretory functions (e.g., hypochlorhydria, pancreatic insufficiency), and immune dysregulation. The diagnostic landscape, while advanced, remains challenging due to symptoms that notoriously overlap with functional disorders. The predominant non-invasive modality—hydrogen and methane breath testing—is hampered by variable protocols and substrate-specific limitations, despite its utility in distinguishing clinical phenotypes such as methane-associated constipation and hydrogen sulfide-linked diarrhea. The historical gold standard, duodenal aspirate culture, remains constrained by its invasiveness and susceptibility to contamination.

Effective clinical management must therefore adopt a structured, multi-component paradigm that extends beyond transient bacterial suppression. The cornerstone strategy integrates targeted, often empirical, antibiotic therapy with rifaximin as a first-line agent, utilizing combination regimens like rifaximin plus neomycin for Intestinal Methanogen Overgrowth (IMO). This is combined with nutritional and dietary support aimed at correcting micronutrient deficiencies—notably vitamin B12 and fat-soluble vitamins—and implementing adjunctive strategies such as the Low FODMAP diet to reduce fermentable substrates. Critically, meticulous management of underlying predisposing conditions is essential to mitigate recurrence. Nevertheless, recurrence rates approaching 44% within nine months underscore the condition’s recalcitrance and the profound influence of persistent underlying pathophysiology.

Future directions must bridge current clinical practice with evolving microbiome science. Key research priorities include the development of standardized diagnostic criteria for breath testing, the validation of novel tools such as telemetric capsules and molecular profiling of duodenal aspirates to advance beyond quantitative thresholds toward dysbiosis-based definitions, and the exploration of personalized, microbiome-informed treatment algorithms. Furthermore, investigating non-antibiotic approaches—including specific probiotics and postbiotics—along with the systemic implications of SIBO, such as its role in tryptophan metabolism and the gut–brain axis, is paramount. Through such integrative and precision-oriented advances, the management of SIBO can progress toward more durable remissions and improved patient outcomes.

## Figures and Tables

**Table 1:** The main clinical manifestation of SIBO.

**Gastrointestinal Symptoms**	**Pathophysiological Mechanism**
Chronic diarrhea	Malabsorption of carbohydrates and bile salts
Steatorrhea	Malabsorption of bile salts
Bloating- Flatulence- Abdominal distension	Hydrogen and methane production from the fermentation of unabsorbed carbohydrates
Abdominal pain	
**Systemic Manifestations**	**Pathophysiological Mechanism**
Weight loss	Hypoproteinemia
Edema, effusions, or ascites	Hypoproteinemia
Anemia	Iron and Vitamin B12 Deficiency
Polyneuropathy	Vitamin B12 deficit
Tetany	Hypocalcemia with Vitamin D deficit
Metabolic bone disease	Hypocalcemia with Vitamin D deficit
Encephalopathy	Increased production of toxins (ammonia, ethanol)

## Data Availability

All data will be available on request to the corresponding author.
